# 2′,10′-Dibromo­spiro­[cylohexane-1,6-di­benzo[*d*,*f*][1,3]dioxepine]

**DOI:** 10.1107/S1600536810012535

**Published:** 2010-04-10

**Authors:** Cheng-Bo Zhang, Hai-Quan Zhang, Qing-Shan Li, Guang-Zhong Xing

**Affiliations:** a State Key Laboratory of Metastable Materials Science and Technology, Yanshan University, Qinhuangdao 066004, People’s Republic of China

## Abstract

In the title compound, C_18_H_16_Br_2_O_2_, the dihedral angle between the aromatic rings is 35.55 (17)° and the cyclohexyl ring adopts a chair-like conformation.  In the crystal, molecules are linked by van der Waals forces.

## Related literature

For background literature concerning title compound, see Dean (1963 ▶); Yang *et al.* (2004 ▶). For details of the synthesis, see Zhang *et al.* (2003 ▶).
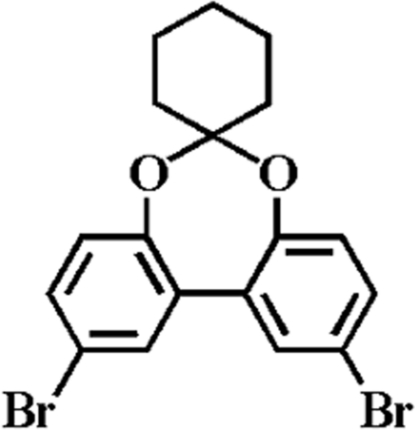


## Experimental

### 

#### Crystal data

C_18_H_16_Br_2_O_2_
*M* *_r_* = 424.13
Orthorhombic, 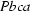

*a* = 17.793 (4) Å
*b* = 10.143 (5) Å
*c* = 18.279 (5) Å
*V* = 3299 (2) Å^3^
*Z* = 8
Mo *K*α radiation
μ = 4.92 mm^−1^
*T* = 290 K
0.13 × 0.12 × 0.11 mm

#### Data collection

Rigaku R-AXIS RAPID diffractometer
Absorption correction: multi-scan (*ABSCOR*; Higashi, 1995 ▶) *T*_min_ = 0.567, *T*_max_ = 0.614
29533 measured reflections
3735 independent reflections
2294 reflections with *I* > 2σ(*I*)
*R*_int_ = 0.088

#### Refinement

*R*[*F* ^2^ > 2σ(*F* ^2^)] = 0.045
*wR*(*F* ^2^) = 0.111
*S* = 1.00
3735 reflections
199 parameters
H-atom parameters constrained
Δρ_max_ = 0.35 e Å^−3^
Δρ_min_ = −0.64 e Å^−3^

### 

Data collection: *RAPID-AUTO* (Rigaku, 1998 ▶); cell refinement: *RAPID-AUTO*; data reduction: *CrystalStructure* (Rigaku/MSC, 2002 ▶); program(s) used to solve structure: *SHELXS97* (Sheldrick, 2008 ▶); program(s) used to refine structure: *SHELXL97* (Sheldrick, 2008 ▶); molecular graphics: *PLATON* (Spek, 2009 ▶); software used to prepare material for publication: *SHELXL97*.

## Supplementary Material

Crystal structure: contains datablocks global, I. DOI: 10.1107/S1600536810012535/ng2752sup1.cif

Structure factors: contains datablocks I. DOI: 10.1107/S1600536810012535/ng2752Isup2.hkl

Additional supplementary materials:  crystallographic information; 3D view; checkCIF report

## Figures and Tables

**Fig. 1.**
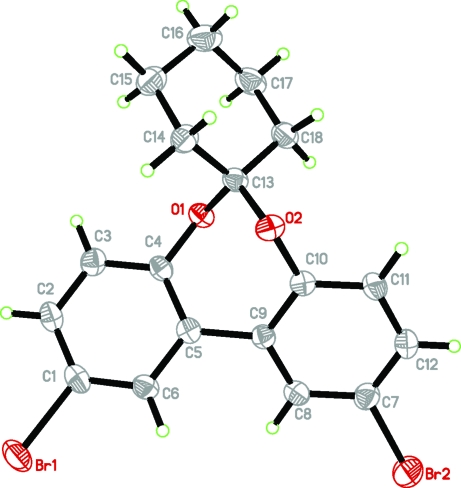
The asymmetric of title compound, with the atom numbering. Displacement ellipsoids of non-H atoms are drawn at the 30% probalility level.

## supplementary crystallographic information

### Comment

Dibenzo[d,f] [1,3] dioxepine derivatives are important seven-member-ring type bridged biphenyl compounds, which proved highly significant for pharmaceutical field (Dean, 1963). Introducing functional group Br on benzene ring of dibenzo[d,f][1,3] dioxepine derivatives can expandthe field of their application, such as photoluminescence, electro-luminescence devices and nonlinear potics etc ( Yang *et al.*, 2004). Herein we present the crysal structure of the title compound.

The molecule structure of title compound, (I), C_18_H_16_Br_2_O_2_, as shown in Fig. 1, all bond lengths and angles are in normal range. In the crystal structure, the six-membered ring formed by C13 to C18 is in the chair-like comformation. The plane of two benzene rings form a dihedral angle of 35.33 (17) °. The crystal packing is stabilized by van der Waals' force.

### Experimental

The title compound was prepared according to the literature (Zhang *et al.*, 2003). Single crystals suitable for X-ray diffraction were prepared by slow evaperation of an ethanol soluion.

### Refinement

Carbon-bound H-atoms were geometrically positioned with C—H = 0.93 and 0.97 Å with U_iso_(H) = 1.2U_eq_(C).

### Crystal data

**Table:**

C_18_H_16_Br_2_O_2_	*F*(000) = 1680
*M**_r_* = 424.13	*D*_x_ = 1.708 Mg m^−^^3^
Orthorhombic, *P**b**c**a*	Mo *K*α radiation, λ = 0.71073 Å
Hall symbol: -P 2ac 2ab	Cell parameters from 3788 reflections
*a* = 17.793 (4) Å	θ = 2.2–54.8°
*b* = 10.143 (5) Å	µ = 4.92 mm^−^^1^
*c* = 18.279 (5) Å	*T* = 290 K
*V* = 3299 (2) Å^3^	Block, white
*Z* = 8	0.13 × 0.12 × 0.11 mm

### Data collection

**Table:**

Rigaku R-AXIS RAPID diffractometer	3735 independent reflections
Radiation source: fine-focus sealed tube	2294 reflections with *I* > 2σ(*I*)
graphite	*R*_int_ = 0.088
ω scans	θ_max_ = 27.5°, θ_min_ = 3.1°
Absorption correction: multi-scan (*ABSCOR*; Higashi, 1995)	*h* = −18→23
*T*_min_ = 0.567, *T*_max_ = 0.614	*k* = −12→12
29533 measured reflections	*l* = −23→23

### Refinement

**Table:**

Refinement on *F*^2^	Primary atom site location: structure-invariant direct methods
Least-squares matrix: full	Secondary atom site location: difference Fourier map
*R*[*F*^2^ > 2σ(*F*^2^)] = 0.045	Hydrogen site location: inferred from neighbouring sites
*wR*(*F*^2^) = 0.111	H-atom parameters constrained
*S* = 1.00	*w* = 1/[σ^2^(*F*_o_^2^) + (0.049*P*)^2^ + 1.6141*P*] where *P* = (*F*_o_^2^ + 2*F*_c_^2^)/3
3735 reflections	(Δ/σ)_max_ = 0.016
199 parameters	Δρ_max_ = 0.35 e Å^−^^3^
0 restraints	Δρ_min_ = −0.64 e Å^−^^3^

### Special details

**Table:**

Experimental. (See detailed section in the paper)
Geometry. All esds (except the esd in the dihedral angle between two l.s. planes) are estimated using the full covariance matrix. The cell esds are taken into account individually in the estimation of esds in distances, angles and torsion angles; correlations between esds in cell parameters are only used when they are defined by crystal symmetry. An approximate (isotropic) treatment of cell esds is used for estimating esds involving l.s. planes.
Refinement. Refinement of *F*^2^ against ALL reflections. The weighted *R*-factor wR and goodness of fit *S* are based on *F*^2^, conventional *R*-factors *R* are based on *F*, with *F* set to zero for negative *F*^2^. The threshold expression of *F*^2^ > σ(*F*^2^) is used only for calculating *R*-factors(gt) etc. and is not relevant to the choice of reflections for refinement. *R*-factors based on *F*^2^ are statistically about twice as large as those based on *F*, and *R*- factors based on ALL data will be even larger.

### Fractional atomic coordinates and isotropic or equivalent isotropic displacement parameters (Å^2^)

**Table:**

	*x*	*y*	*z*	*U*_iso_*/*U*_eq_	
Br1	1.04490 (3)	0.06701 (5)	0.35791 (2)	0.07070 (18)	
Br2	0.85968 (3)	0.22102 (6)	0.73751 (2)	0.0790 (2)	
C1	1.0023 (2)	0.2318 (4)	0.38479 (19)	0.0504 (10)	
C2	1.0211 (2)	0.3410 (4)	0.3440 (2)	0.0509 (10)	
H2	1.0543	0.3341	0.3049	0.061*	
C3	0.9896 (2)	0.4612 (4)	0.36234 (18)	0.0468 (9)	
H3	1.0008	0.5357	0.3348	0.056*	
C4	0.94151 (19)	0.4708 (4)	0.42156 (18)	0.0384 (8)	
C5	0.92258 (19)	0.3603 (4)	0.46297 (17)	0.0393 (8)	
C6	0.9533 (2)	0.2386 (4)	0.44328 (18)	0.0447 (9)	
H6	0.9409	0.1629	0.4693	0.054*	
C7	0.8410 (2)	0.3224 (4)	0.65214 (18)	0.0487 (9)	
C8	0.8863 (2)	0.3024 (4)	0.59131 (18)	0.0436 (9)	
H8	0.9245	0.2400	0.5928	0.052*	
C9	0.87442 (18)	0.3757 (4)	0.52831 (17)	0.0375 (8)	
C10	0.81592 (19)	0.4670 (4)	0.52830 (17)	0.0399 (8)	
C11	0.7721 (2)	0.4878 (4)	0.5897 (2)	0.0485 (9)	
H11	0.7339	0.5504	0.5887	0.058*	
C12	0.7850 (2)	0.4158 (4)	0.65240 (19)	0.0532 (10)	
H12	0.7564	0.4302	0.6942	0.064*	
C13	0.84221 (18)	0.6288 (4)	0.43511 (18)	0.0402 (8)	
C14	0.8204 (2)	0.6406 (4)	0.35503 (17)	0.0488 (9)	
H14A	0.7663	0.6509	0.3512	0.059*	
H14B	0.8343	0.5603	0.3295	0.059*	
C15	0.8591 (2)	0.7583 (5)	0.3188 (2)	0.0610 (12)	
H15A	0.9128	0.7420	0.3163	0.073*	
H15B	0.8406	0.7681	0.2692	0.073*	
C16	0.8450 (3)	0.8833 (5)	0.3606 (2)	0.0709 (13)	
H16A	0.7919	0.9043	0.3588	0.085*	
H16B	0.8723	0.9551	0.3377	0.085*	
C17	0.8696 (3)	0.8706 (4)	0.4399 (2)	0.0634 (11)	
H17A	0.9236	0.8582	0.4420	0.076*	
H17B	0.8575	0.9511	0.4660	0.076*	
C18	0.8310 (2)	0.7552 (4)	0.4764 (2)	0.0507 (10)	
H18A	0.8506	0.7451	0.5256	0.061*	
H18B	0.7776	0.7735	0.4802	0.061*	
O1	0.91933 (12)	0.5946 (2)	0.44369 (12)	0.0406 (6)	
O2	0.79528 (13)	0.5265 (3)	0.46336 (12)	0.0439 (6)	

### Atomic displacement parameters (Å^2^)

**Table:**

	*U*^11^	*U*^22^	*U*^33^	*U*^12^	*U*^13^	*U*^23^
Br1	0.0773 (3)	0.0523 (3)	0.0825 (3)	0.0132 (2)	0.0174 (2)	−0.0179 (2)
Br2	0.1021 (4)	0.0865 (4)	0.0482 (3)	0.0256 (3)	0.0059 (2)	0.0173 (2)
C1	0.050 (2)	0.049 (3)	0.052 (2)	0.0083 (19)	−0.0017 (17)	−0.0152 (18)
C2	0.050 (2)	0.053 (3)	0.049 (2)	0.0007 (19)	0.0078 (17)	−0.0141 (19)
C3	0.042 (2)	0.049 (3)	0.049 (2)	−0.0094 (18)	0.0049 (16)	−0.0013 (17)
C4	0.0355 (19)	0.036 (2)	0.0436 (18)	−0.0013 (15)	−0.0019 (14)	−0.0083 (15)
C5	0.0358 (19)	0.041 (2)	0.0407 (17)	−0.0019 (16)	−0.0029 (14)	−0.0054 (15)
C6	0.047 (2)	0.037 (2)	0.051 (2)	0.0000 (18)	0.0004 (16)	−0.0026 (16)
C7	0.055 (2)	0.050 (3)	0.0416 (19)	−0.0012 (19)	0.0015 (16)	0.0006 (17)
C8	0.045 (2)	0.040 (2)	0.0448 (19)	0.0040 (17)	−0.0025 (15)	−0.0028 (16)
C9	0.0367 (19)	0.035 (2)	0.0406 (18)	−0.0023 (16)	0.0005 (14)	−0.0033 (15)
C10	0.039 (2)	0.040 (2)	0.0413 (18)	−0.0010 (16)	−0.0017 (14)	0.0008 (15)
C11	0.039 (2)	0.050 (3)	0.057 (2)	0.0081 (18)	0.0087 (16)	0.0004 (18)
C12	0.055 (2)	0.059 (3)	0.046 (2)	0.007 (2)	0.0129 (17)	−0.0014 (18)
C13	0.033 (2)	0.037 (2)	0.0505 (19)	0.0008 (16)	0.0029 (14)	0.0023 (16)
C14	0.047 (2)	0.054 (3)	0.046 (2)	0.0001 (19)	0.0004 (16)	0.0043 (17)
C15	0.052 (2)	0.072 (4)	0.058 (2)	0.004 (2)	0.0043 (18)	0.018 (2)
C16	0.062 (3)	0.057 (3)	0.094 (3)	0.003 (2)	0.009 (2)	0.025 (3)
C17	0.065 (3)	0.039 (3)	0.086 (3)	0.001 (2)	0.007 (2)	−0.003 (2)
C18	0.050 (2)	0.044 (3)	0.058 (2)	0.0050 (19)	0.0041 (17)	−0.0028 (18)
O1	0.0348 (13)	0.0354 (16)	0.0516 (13)	−0.0005 (11)	−0.0001 (10)	−0.0037 (11)
O2	0.0379 (14)	0.0448 (17)	0.0489 (13)	−0.0042 (11)	−0.0044 (11)	0.0071 (11)

### Geometric parameters (Å, °)

**Table:**

Br1—C1	1.900 (4)	C11—H11	0.9300
Br2—C7	1.898 (4)	C12—H12	0.9300
C1—C2	1.377 (6)	C13—O1	1.424 (4)
C1—C6	1.381 (5)	C13—O2	1.428 (4)
C2—C3	1.382 (6)	C13—C18	1.501 (5)
C2—H2	0.9300	C13—C14	1.519 (5)
C3—C4	1.384 (5)	C14—C15	1.530 (5)
C3—H3	0.9300	C14—H14A	0.9700
C4—O1	1.376 (4)	C14—H14B	0.9700
C4—C5	1.394 (5)	C15—C16	1.502 (6)
C5—C6	1.398 (5)	C15—H15A	0.9700
C5—C9	1.478 (5)	C15—H15B	0.9700
C6—H6	0.9300	C16—C17	1.521 (6)
C7—C12	1.374 (5)	C16—H16A	0.9700
C7—C8	1.388 (5)	C16—H16B	0.9700
C8—C9	1.387 (5)	C17—C18	1.512 (6)
C8—H8	0.9300	C17—H17A	0.9700
C9—C10	1.394 (5)	C17—H17B	0.9700
C10—O2	1.381 (4)	C18—H18A	0.9700
C10—C11	1.383 (5)	C18—H18B	0.9700
C11—C12	1.379 (5)		
			
C2—C1—C6	122.2 (4)	O1—C13—C18	106.3 (3)
C2—C1—Br1	118.1 (3)	O2—C13—C18	111.1 (3)
C6—C1—Br1	119.7 (3)	O1—C13—C14	111.8 (3)
C1—C2—C3	118.7 (3)	O2—C13—C14	104.8 (3)
C1—C2—H2	120.7	C18—C13—C14	112.6 (3)
C3—C2—H2	120.7	C13—C14—C15	111.3 (3)
C4—C3—C2	120.2 (4)	C13—C14—H14A	109.4
C4—C3—H3	119.9	C15—C14—H14A	109.4
C2—C3—H3	119.9	C13—C14—H14B	109.4
O1—C4—C3	118.2 (3)	C15—C14—H14B	109.4
O1—C4—C5	120.3 (3)	H14A—C14—H14B	108.0
C3—C4—C5	121.2 (3)	C16—C15—C14	111.3 (3)
C4—C5—C6	118.4 (3)	C16—C15—H15A	109.4
C4—C5—C9	119.6 (3)	C14—C15—H15A	109.4
C6—C5—C9	121.9 (3)	C16—C15—H15B	109.4
C1—C6—C5	119.4 (4)	C14—C15—H15B	109.4
C1—C6—H6	120.3	H15A—C15—H15B	108.0
C5—C6—H6	120.3	C15—C16—C17	111.4 (4)
C12—C7—C8	121.6 (3)	C15—C16—H16A	109.3
C12—C7—Br2	119.8 (3)	C17—C16—H16A	109.3
C8—C7—Br2	118.5 (3)	C15—C16—H16B	109.3
C9—C8—C7	119.9 (3)	C17—C16—H16B	109.3
C9—C8—H8	120.0	H16A—C16—H16B	108.0
C7—C8—H8	120.0	C18—C17—C16	110.8 (4)
C8—C9—C10	118.0 (3)	C18—C17—H17A	109.5
C8—C9—C5	121.8 (3)	C16—C17—H17A	109.5
C10—C9—C5	120.2 (3)	C18—C17—H17B	109.5
O2—C10—C11	118.7 (3)	C16—C17—H17B	109.5
O2—C10—C9	119.3 (3)	H17A—C17—H17B	108.1
C11—C10—C9	121.5 (3)	C13—C18—C17	112.2 (3)
C12—C11—C10	120.0 (3)	C13—C18—H18A	109.2
C12—C11—H11	120.0	C17—C18—H18A	109.2
C10—C11—H11	120.0	C13—C18—H18B	109.2
C7—C12—C11	118.9 (3)	C17—C18—H18B	109.2
C7—C12—H12	120.5	H18A—C18—H18B	107.9
C11—C12—H12	120.5	C4—O1—C13	117.8 (3)
O1—C13—O2	110.3 (3)	C10—O2—C13	118.2 (3)
